# Beyond Visualization: Advanced Imaging, Theragnostics and Biomarker Integration in Urothelial Bladder Cancer

**DOI:** 10.3390/cancers17193261

**Published:** 2025-10-08

**Authors:** Eduardo Albers Acosta, Lira Pelari Mici, Carlos Márquez Güemez, Clara Velasco Balanza, Manuel Saavedra Centeno, Marta Pérez Pérez, Guillermo Celada Luis, Cristina Quicios Dorado, José Daniel Subiela, Rodrigo España Navarro, Patricia Toquero Diez, Nuria Romero Laorden, Luis San José Manso

**Affiliations:** 1 Department of Urology, Hospital Universitario de La Princesa, IIS Princesa, Universidad Autónoma de Madrid, 28006 Madrid, Spain; lira.pelari@salud.madrid.org (L.P.M.); carlos.marquez.externo@salud.madrid.org (C.M.G.); clara.velasco@salud.madrid.org (C.V.B.); manuel.saavedra@salud.madrid.org (M.S.C.); marta.perezp.externo@salud.madrid.org (M.P.P.); guillermo.celada@salud.madrid.org (G.C.L.); cristina.quicios@salud.madrid.org (C.Q.D.); lsanjose.hcsc@salud.madrid.org (L.S.J.M.); 2 Department of Urology, Hospital Universitario Ramón y Cajal, IRYSIS, Universidad de Alcalá, 28034 Madrid, Spain; josedaniel.subiela@salud.madrid.org; 3 Department of Urology, Hospital Regional Universitario de Málaga, TAUS CLINICS, 29010 Málaga, Spain; rodrigo.espana.sspa@juntadeandalucia.es; 4 Department of Medical Oncology, Hospital Universitario de La Princesa, IIS Princesa, Universidad Autónoma de Madrid, 28006 Madrid, Spain; patricia.toquero@salud.madrid.org (P.T.D.); nromerol@salud.madrid.org (N.R.L.)

**Keywords:** urothelial carcinoma, artificial intelligence, radiotracer, cystoscopy, immuno-PET, liquid biopsy, circulating tumor cells, urinary biomarkers

## Abstract

**Simple Summary:**

Bladder cancer frequently recurs and may progress. Emerging tools—such as positron emission tomography (PET) tracers beyond fluorodeoxyglucose (FDG), artificial intelligence (AI)-assisted cystoscopy, theragnostics, and liquid biopsy biomarkers—are reshaping how this disease is detected, staged, and treated. This narrative review summarizes recent advances and discusses their clinical implications. Integrating molecular imaging, AI, and biomarker data holds the potential to deliver more precise and genuinely personalized care.

**Abstract:**

**Background/Objectives:** Bladder cancer is characterized by high recurrence and progression rates, posing a challenge to current diagnostic and treatment strategies. This review aims to provide a comprehensive overview of emerging technologies, including novel PET tracers, AI-assisted cystoscopy, theragnostics, and molecular biomarkers. **Methods**: We performed a narrative review of the recent literature focusing on innovations in imaging, AI, theragnostics, and biomarker research relevant to bladder cancer diagnosis and management. **Results**: Several novel PET tracers, such as 68Ga-PSMA and fibroblast activation protein inhibitor (FAPI), demonstrated potential in improving detection sensitivity. AI-enhanced cystoscopy has shown promise in real-time lesion detection, while theragnostic agents enable combined diagnostic and therapeutic applications. Advances in molecular biomarkers, including circulating Tumor DNA (ctDNA) and gene expression signatures, offer new avenues for patient stratification and monitoring. **Conclusions**: Integration of advanced imaging, AI, theragnostics, and biomarker analysis may transform bladder cancer management, supporting personalized and more effective care strategies.

## 1. Introduction

Bladder cancer is the tenth most common malignancy worldwide, with approximately 570,000 new cases and 210,000 deaths each year [1]. Despite improvements in surgical and systemic therapies, recurrence and progression remain major clinical challenges, reflecting the biological heterogeneity of the disease [2]. Conventional diagnostic tools, such as computed tomography (CT) and white-light cystoscopy (WLC), are limited in sensitivity, specificity, and tumor characterization, particularly for small or flat lesions, highlighting the need for innovative, non-anatomical approaches [3].

Recent advances in molecular imaging, theragnostic agents, artificial intelligence (AI)-assisted cystoscopy, and biomarker research are reshaping the diagnostic and therapeutic landscape of urothelial carcinoma [4]. These developments enable a deeper understanding of tumor biology, support personalized treatment strategies, and may help overcome the limitations of conventional imaging. This review summarizes key innovations in imaging, theragnostics, and biomarker integration, providing a concise, practice-oriented overview of how these technologies may advance precision oncology in bladder cancer.

## 2. Materials and Methods

This work is a narrative review aimed at summarizing the latest developments in molecular imaging, theragnostics, and biomarker integration in urothelial cancer. A structured literature search was performed in PubMed, Embase, and the Cochrane Library up to May 2025 using combinations of the keywords “bladder cancer”, “urothelial carcinoma”, “molecular imaging”, “theragnostics”, “PET/CT”, “artificial intelligence”, and “liquid biopsy”. Articles published in English were included if they reported relevant clinical, translational, or imaging-based findings. Priority was given to peer-reviewed original studies, meta-analyses, and high-quality reviews. References for selected papers were also screened for additional relevant publications.

As this was a narrative (non-systematic) review, no formal quantitative analysis or quality assessment was performed. The focus was to provide a critical and integrated synthesis of the most relevant and recent evidence to support clinical and translational understanding.

Two reviewers screened studies independently and resolved discrepancies by consensus. Data extracted comprised study design, population, modality/algorithm, tracers or biomarkers, main outcomes, and clinical implications. Owing to study heterogeneity, findings were synthesized narratively. Key methodological elements and evidence strength are summarized in Table 1.

## 3. Results

### 3.1. Advances in Molecular Imaging and Emerging PET Tracers in Bladder Cancer

Positron emission tomography with [^18^F] fluorodeoxyglucose (FDG) has been a cornerstone in many solid malignancies, but its role in bladder cancer is limited by urinary excretion and low specificity in adjacent tissues [5]. Recent advances have focused on developing radiopharmaceuticals that improve tumor characterization and allow the identification of therapeutic targets.

#### 3.1.1. Fibroblast Activation Protein Inhibitors (FAPI)

Among novel tracers, fibroblast activation protein inhibitor (FAPI) tracers have gained attention due to high tumor uptake and low background signal. In bladder cancer, Gallium-68-labeled FAPI PET/CT has demonstrated utility in lymphatic and haematogenous staging of high-grade tumors, with superior sensitivity for subcentimeter metastases compared with conventional imaging, although urinary excretion still limits primary-tumor assessment [6,7].

Compared with FDG, FAPI consistently shows higher tumor-to-background ratios (5.3–5.6 vs. 1.9–1.95; *p* = 0.001) and increased detection rates of metastases (~30%), particularly in lymph nodes, lungs and peritoneum, including lesions missed by FDG or CT [8,9]. In a prospective pilot study with [^68^Ga]FAPI-2286, tracer uptake exceeded FDG (SUVmax 9.9 ± 3.4 vs. 4.2 ± 1.9; *p* < 0.0001), correlated with lesion size (*p* = 0.001), and altered surgical planning in three cases [10]. Preoperatively, [^68^Ga]FAPI-46 PET/CT achieved 95.2% specificity and 93% negative predictive value for nodal staging, supporting its role in tailoring lymphadenectomy [11]. A recent systematic review confirmed these advantages for small nodal and peritoneal metastases while emphasizing the need for optimized protocols for primary-tumor evaluation [7]. Overall, FAPI tracers show consistently higher lesion detectability than FDG and could become valuable tools for nodal and peritoneal staging once larger, standardized studies confirm their performance.

#### 3.1.2. Alternative Modalities and Computational Tools

Beyond tracer innovation, hybrid imaging and AI-based analysis are expanding diagnostic capabilities in bladder cancer.

1. [^11^C]-Acetate PET/Magnetic Resonance Imaging (MRI): The ACEBIB trial evaluated Carbon-11-labeled acetate ([^11^C]-acetate) PET/MRI as an alternative tracer for staging and treatment monitoring in bladder cancer. [^11^C]-acetate targets lipid metabolism, which is upregulated in many tumors, and offers the advantage of minimal urinary excretion compared to FDG, reducing interference in bladder imaging. In this multicenter study, the technique achieved 100% sensitivity for detecting muscle-invasive disease and showed reasonable correlation with response to neoadjuvant chemotherapy. However, its sensitivity for nodal metastases was limited (20%), underscoring the need for further validation before clinical adoption [12].
2. AI applied to PET/FDG: To improve the limited accuracy of FDG PET/CT in nodal staging, machine learning has been explored as a complementary tool. In one study, a random forest model was developed using three objective parameters—Maximum Standardized Uptake Value (SUVmax) of the most avid lymph node, product of diameters of the largest node, and primary tumor size. This model achieved higher diagnostic accuracy than expert consensus in the training cohort (area under curve (AUC) 0.87 vs. 0.73; *p* = 0.048). Although performance did not hold in the independent validation set (AUC 0.59 vs. 0.64; *p* = 0.54), interrater agreement was excellent (kappa = 0.66), suggesting that AI-based approaches could provide reproducible, standardized support for lymph node assessment in muscle-invasive bladder cancer [13].

These findings come mainly from small, single-center studies; prospective multicenter validation will be needed before these alternative tracers and AI tools can be adopted in routine practice.

#### 3.1.3. Emerging Molecular Targets for Imaging

Efforts to identify novel imaging targets in bladder cancer have evaluated markers such as Vascular Endothelial Growth Factor (VEGF), Epidermal Growth Factor Receptor (EGFR), and Prostate-Specific Membrane Antigen (PSMA) through immunohistochemistry (IHC) of metastatic lymph nodes. Expression was quantified using a composite immunostaining score (range 0–12) based on staining intensity and proportion of positive cells. VEGF showed moderate to high expression in metastatic nodes (median score 8/12), with acceptable contrast compared to tumor-negative nodes (mean ratio 1.3 ± 0.7), supporting its feasibility for targeted imaging. In contrast, EGFR and PSMA demonstrated only weak staining (median scores 2–3), limiting their potential as imaging targets [14]. VEGF appears feasible for targeted imaging, whereas EGFR and PSMA show limited potential; all remain to be validated in bladder cancer-specific trials.

#### 3.1.4. Nectin-4-Targeted Imaging

As an adhesion molecule overexpressed in urothelial carcinoma, Nectin-4 is of particular interest. In a first-in-human trial, the novel tracer ^68^Ga-N188 demonstrated strong correlation between PET uptake and IHC expression (SUVmax 8.3 ± 2.4 for strong positive vs. 3.7 ± 1.1 for weak and 1.9 ± 0.4 for negative cases; *p* < 0.001). ROC analysis confirmed high predictive value, with AUC 0.967, sensitivity 88.1%, and specificity 100% at an SUVmax threshold of 2.85 [15]. However, these encouraging data are still limited to a first-in-human pilot; larger bladder cancer-specific studies are needed to confirm clinical utility.

Table 2 summarizes recent advances in radiopharmaceuticals designed to improve tumor characterization and identify therapeutic targets.

This table shows novel tracers that improve sensitivity for detecting metastatic spread and reduce urinary interference, yet heterogeneity across studies and the lack of large prospective trials still limit their integration into routine clinical practice.

### 3.2. Enhanced Cystoscopic Evaluation Through AI and Optical Imaging

White-light cystoscopy (WLC) remains the gold standard for bladder cancer diagnosis. While it offers good sensitivity for papillary tumors, its performance in detecting carcinoma in situ and small or flat lesions is suboptimal, contributing to incomplete resections and underdiagnosis [16,17]. Up to 10–20% of tumors may be missed with WLC [18], and incomplete Transurethral Resection of Bladder Tumors (TURBT) has been reported in nearly 50% of cases [19], favoring early recurrence and progression.

#### 3.2.1. Advances in Optical Imaging and the Role of AI

To address these limitations, enhanced optical modalities (imaging techniques that augment conventional white light cystoscopy) have been developed. The most established examples are fluorescence cystoscopy and narrow-band imaging (NBI), both of which improve the visualization of inconspicuous lesions and show superior sensitivity compared to WLC alone. Evidence from two meta-analyses reported sensitivity rates of approximately 90% for fluorescence and NBI, compared to around 75% for WLC [18,20]. However, clinical uptake remains limited due to logistical, economic, and training barriers. More recently, AI has emerged as a complementary tool to improve detection, segmentation, and prognostication, particularly in non-muscle-invasive bladder cancer (NMIBC), where complete TURBT and early diagnosis are critical [3,4,19,20,21].

#### 3.2.2. AI-Based Detection and Real-Time Imaging

Deep learning applied to cystoscopy has markedly improved detection accuracy. Models trained on blue-light images have achieved sensitivities above 91% and specificities over 77% for NMIBC during TURBT [22]. Some groups have gone further by combining optical techniques such as fluorescence and diffuse reflectance spectroscopy with machine learning, allowing real-time characterization of tumor tissue, achieving sensitivity and specificity of 78% and 91%, respectively [23].

Convolutional neural networks (CNNs), which automatically learn image patterns, have shown consistent ability to discriminate tumors from benign lesions, with sensitivities ranging from 85 to 90% and specificities from 89 to 94% in retrospective analyses [24,25]. More advanced architectures, including high-resolution CNN models, have achieved even higher performance, with sensitivities of 91.6–94.8% and precisions of 91.3–94.4%, particularly improving detection of flat lesions such as carcinoma in situ [26]. Their robustness has been confirmed in multicenter evaluations, where one system reported an AUC of 0.96 [27], and another (INF-M01) achieved 97.3% sensitivity, 92.1% specificity, and 93.4% overall accuracy in routine clinical practice, effectively serving as a reliable second observer [28]. These results show that deep learning and combined optical–AI approaches can reach diagnostic accuracies comparable to expert observers and bring real-time tumor characterization within reach of routine TURBT.

#### 3.2.3. Tumor Segmentation and Dual-Function Models

Precise visualization of tumor margins is essential for complete resection. AI-based segmentation models enhance image contrast and boundary clarity, allowing more accurate identification of tumor borders, even in early disease, with reported accuracies of around 91% in retrospective analyses [29]. Systems such as U-Net have shown high precision in outlining lesions during cystoscopy [30], while simplified versions designed for lower computational demands make real-time use feasible in standard clinical settings [31]. More advanced dual-function models can not only delineate the tumor but also classify its type (normal, papillary, or flat), reaching over 91% accuracy. Their boundary detection closely matches expert performance (Intersection over Union (IoU) 0.833; binary accuracy 95.1%), and early evidence suggests they could also support automated tumor grading in a single step [32]. These advances suggest that AI-based segmentation and dual-function models could soon support surgeons in achieving more complete resections and standardized tumor classification in routine practice.

#### 3.2.4. Grading, Biological Behavior, and Real-Time Augmentation

AI systems are also being developed to predict tumor grade and biological behavior directly from cystoscopic images [33]. By analyzing color and texture features, these models have achieved performance comparable to experienced clinicians, with one study reporting a balanced accuracy of 81%, sensitivity of 88%, specificity of 74%, and an AUC of 92% [34,35]. Beyond static classification, AI is increasingly integrated into live cystoscopy, where real-time augmentation platforms enhance visualization under suboptimal lighting, reduce false positives, raise specificity to as high as 98.8%, and minimize interobserver variability [36,37,38]. Altogether, these advances show that AI can help make cystoscopy more accurate and consistent in everyday practice.

An overview of studies combining AI with cystoscopy is presented in Table 3.

This table illustrates the rapid evolution from basic detection systems to advanced segmentation and grading approaches, as well as the methodological diversity that still limits clinical translation.

### 3.3. Experimental Theragnostic Approaches in Bladder Cancer

#### 3.3.1. From Immunotherapy to Immuno-Theragnostics

Bladder cancer, characterized by high mutational load and immune evasion, has seen immune checkpoint inhibitors (ICIs) such as anti-PD-1 and anti-PD-L1 become essential in advanced disease or cisplatin-ineligible patients [39,40,41,42,43]. Building on this, theragnostic strategies now aim to visualize and treat immune-related targets simultaneously. Immuno-PET allows in vivo imaging of PD-L1 and CD8+ T cells, enabling non-invasive patient selection, monitoring of immune dynamics, and anticipation of response [44,45]. To date, most of these approaches remain preclinical or exploratory in solid tumors other than bladder cancer, with no disease-specific prospective human trials yet available. These early data are promising, but further bladder cancer-specific validation will be needed.

#### 3.3.2. Immune-Related Targets

ICIs are central to NMIBC unresponsive to Bacillus Calmette–Guérin (BCG) and remain integral in muscle invasive bladder cancer (MIBC) and advanced stages [41,42,43]. Response rates are higher in patients with elevated PD-L1 expression, though long-term durability remains a challenge [46,47,48]. For patients refractory to both platinum chemotherapy and ICIs, theragnostic applications are under investigation, linking imaging tracers to therapeutic agents directed against PD-1, PD-L1, and other tumor-associated markers such as Nectin-4 and TROP-2 [49,50]. Radiolabeled antibodies and small-molecule tracers for PD-L1 are in early-phase immuno-PET trials, offering real-time, non-invasive quantification of expression, though none are currently bladder cancer-specific [51,52]. Thus, while conceptually attractive, these strategies remain speculative in urothelial carcinoma.

#### 3.3.3. Nectin-4 and TROP-2: Companion Diagnostics and Antibody-Drug Conjugates

Nectin-4 and TROP-2 are clinically validated therapeutic targets. The Antibody-drug conjugates enfortumab vedotin (anti-Nectin-4) and sacituzumab govitecan (anti-TROP-2) have achieved objective response rates of 31–44% in advanced or heavily pretreated bladder cancer, including patients post-ICI therapy [53,54,55]. Evidence is strongest in advanced MIBC, with no robust support yet for NMIBC or long-term outcomes from phase III studies. Expression patterns support precision medicine: Nectin-4 is most abundant in squamous and plasmacytoid variants, while PD-L1 is more common in sarcomatoid tumors [40,56]. In parallel, PET tracers targeting Nectin-4 have demonstrated strong correlation with immunohistochemistry, but these findings are still limited to preclinical work and exploratory human data, with no validated bladder cancer-specific trials available.

### 3.4. Liquid Biopsy and Circulating Biomarkers

Liquid biopsy offers minimally invasive, real-time monitoring through circulating tumor cells (CTCs), circulating tumor DNA (ctDNA), Ribonucleic Acid (RNAs), and proteins [57].

#### 3.4.1. CTCs

The detection of CTCs increases with disease burden, reported in 10.3% of NMIBC, 28.6% of MIBC, 40% of nodal disease, and up to 75% of metastatic cases [58]. Reviews and meta-analyses confirm their correlation with tumor stage and grade, with pooled sensitivity of 35% and specificity of 97% [59,60].

Prognostic studies further support their relevance. A 2025 meta-analysis demonstrated that CTC positivity after TURBT significantly predicted recurrence, with a hazard ratio for progression from T1 to T2 of 3.36 (95% CI: 2.68–3.25) [61]. In a prospective cohort of 84 patients, elevated postoperative CTCs correlated with high Ki-67 expression and poorer survival outcomes [62]. Similarly, in a multicenter study of 273 radical cystectomy patients, CTC-negative patients experienced lower relapse and cancer-specific mortality, while CTC-positive patients who received neoadjuvant therapy achieved improved survival, highlighting their potential role as predictive biomarkers [63]. These studies indicate that CTC detection reflects disease burden and progression risk, and may help identify patients most likely to benefit from perioperative therapy.

#### 3.4.2. ctDNA

Because ctDNA turns over rapidly in plasma, it can capture short-term changes in tumor burden and treatment effect, supporting real-time monitoring [64]. In MIBC, a 2025 systematic review showed that preoperative ctDNA positivity was associated with shorter recurrence-free survival and more advanced disease, while postoperative detection correlated with reduced disease-free Survival (DFS) and higher recurrence. Importantly, ctDNA clearance during neoadjuvant chemotherapy (NAC) aligned with treatment response, and longitudinal ctDNA dynamics mirrored efficacy during immunotherapy [65]. A 2024 meta-analysis (16 studies; 1725 patients) reported that elevated baseline ctDNA predicted worse DFS (Hazard Ratio (HR) = 6.26; 95% CI: 3.71–10.58) and overall survival (OS) (HR = 4.23; 95% CI: 2.72–6.57); conversely, on-treatment declines in ctDNA were associated with improved survival, and specific alterations (e.g., FGFR3 mutations) further refined prognostic stratification [66]. Taken together, ctDNA levels and dynamics provide a real-time measure of tumor burden and treatment response, offering a powerful prognostic and monitoring tool once validated in larger cohorts.

#### 3.4.3. RNA Biomarkers

1. mRNA-based assays: Several commercial urine-based assays using messenger RNA (mRNA) expression have demonstrated robust diagnostic performance in bladder cancer. The CxBladder Monitor (genes: CDK1, CXCR2, HOXA13, IGFBP5, MDK) reported 91% sensitivity, 96% negative predictive value, and an AUC of 0.73. The Xpert BC test (ABL1, ANXA10, CRH, IGF2, UPK1B) achieved 84% sensitivity and 91% specificity (AUC 0.872). Similarly, UROBEST reached 80% sensitivity and 94% specificity (AUC 0.91), while the Oncocyte 43-gene panel achieved 90% sensitivity and 82.5% specificity (AUC 0.91) [67]. Together, these data highlight that mRNA assays are among the most advanced biomarker tools, with some already available for clinical use.
2. miRNAs: MicroRNAs (miRNAs), small regulatory RNAs detectable in urine, have also been explored extensively. A 2022 systematic review of 25 studies (*N* = 4054) identified multiple miRNA candidates with diagnostic potential, though inter-study variability limited consistent signatures [68]. More recently, a 2024 meta-analysis focusing on miR-143 reported sensitivity 0.80, specificity 0.85, and AUC 0.88, underscoring its potential as a reliable non-invasive marker [69]. It shows promising accuracy but still needs consistent validation.
3. lncRNAs: Long non-coding RNAs (lncRNAs), particularly when carried in urinary exosomes, have also been evaluated. A 2021 meta-analysis (1883 bladder cancer patients vs. 1721 controls) demonstrated pooled sensitivity 0.74, specificity 0.76, and AUC 0.83. Importantly, panels of multiple lncRNAs outperformed single markers (AUC 0.86 vs. 0.81), supporting their clinical utility as part of multiparametric approaches [70].
4. Emerging Biomarkers and Multi-Omic Signatures: In addition to circulating tumor DNA (ctDNA) and circulating tumor cells (CTCs), several new biomarkers are under investigation. These include circular RNAs (circRNAs), the RNA demethylase FTO (fat mass and obesity-associated protein, an epitranscriptomic regulator), and distinct protein or peptide signatures—all linked to bladder cancer biology but still requiring further validation [71]. At the genomic level, mutation panels covering genes such as TP53, FGFR3, ERBB2, PIK3CA, ARID1A and FGFR3-ADD1 fusions may help detect minimal residual disease and monitor relapse. These emerging biomarkers could improve early detection and monitoring of bladder cancer once validated.

To improve detection of minimal residual disease, two main approaches are being explored. Tumor-informed panels, designed from each patient’s own tumor sequence, provide higher sensitivity and specificity. In contrast, tumor-agnostic panels use predefined gene sets without prior sequencing of the individual tumor; they are faster and easier to deploy but more prone to false positives [72].

Similarly, fragmentomics refers to the analysis of the size and pattern of circulating DNA fragments. Approaches such as EPIC-seq use these fragmentation patterns to infer gene expression from ctDNA, showing promise even in patients with low tumor burden.

Because bladder cancer is highly heterogeneous, a single biomarker is often not enough. Multi-omic approaches combine different types of biological information such as genetic changes (genomics), gene activity (transcriptomics), proteins (proteomics) and epigenetic marks (epigenomics) to give a more complete picture of the disease and improve risk stratification. Using this kind of analysis, a 2025 study identified sex-specific prognostic signatures: RAD51C, COL22A1 and COL5A2 in females, and DAXX, IKBKB, PDGFRA and PPARG in males, many of which are linked to immune pathways [73].

Other combined approaches also show promise. For example, Surface-Enhanced Raman Spectroscopy (SERS) with urinary miRNAs increased diagnostic accuracy (AUC 0.92–0.97) [74]. Urinary proteins such as elastase, S100A12, p53, and kallikrein-6 have been correlated with tumor stage and immune signaling [75]. Similarly, DNA methylation assays of urinary exfoliated cells achieved 89% sensitivity and 92% specificity for bladder cancer detection [76].

Importantly, systematic reviews confirm that combining biomarkers enhances diagnostic performance, particularly in early-stage or recurrent disease. This principle is being tested in the NEO-BLAST trial, which evaluates a multimodal strategy combining urinary tumor DNA (utDNA), plasma ctDNA, imaging, and repeat TURBT to guide bladder preservation after NAC. Patients with negative ctDNA/utDNA and complete response are followed under active surveillance, while discordant profiles may proceed to cystectomy [77].

Altogether, these findings underscore the growing relevance of multi-marker and multi-omic approaches, which may overcome the limitations of single assays and move clinical practice closer to true precision oncology in bladder cancer.

#### 3.4.4. Integration with Imaging

Radiomics converts imaging data into quantifiable features; while radiogenomics links these patterns to genomic and transcriptomic profiles. A 2025 review found radiogenomic nomograms predicted outcomes with sensitivity 0.83 and specificity 0.815 [78]. Integration with liquid biopsy adds further predictive value: in advanced urothelial carcinoma, changes in ctDNA allele fraction (ΔaVAF) predicted progression 92 days before radiology and improved survival estimation [79].

An overview of landmark studies in biomarkers and imaging integration appears in Table 4.

This table summarizes recent advances aimed at improving diagnostic accuracy, prognostic assessment, and therapeutic decision-making. Most studies highlight the potential of ctDNA and CTCs as clinically relevant biomarkers that could support more personalized and precise management of patients with bladder cancer.

## 4. Discussion

**Figure 1:**
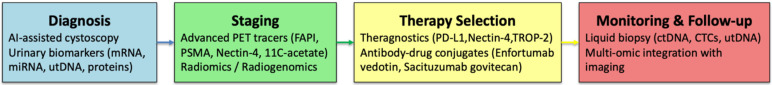
Flowchart illustrates the integration of advanced imaging, AI, theragnostics, and biomarkers across the diagnostic–therapeutic pathway in bladder cancer.

Molecular imaging in bladder cancer is undergoing a paradigm shift with the development of novel PET tracers, computational tools, and immune-targeted theragnostics. To illustrate the complementary role of these modalities, we have added a summary flowchart (Figure 1) that outlines the diagnostic–therapeutic pathway in bladder cancer, integrating AI-assisted cystoscopy, molecular imaging, theragnostics, and liquid biopsy.

The limitations of FDG in bladder imaging (primarily its urinary excretion) have driven the exploration of alternative tracers such as FAPI. These radiopharmaceuticals provide improved tumor-to-background ratios and enhanced sensitivity for small-volume metastases, particularly in lymph nodes and peritoneal deposits [6,7,8,9]. Beyond improving staging, FAPI-based PET may influence treatment planning by refining surgical strategies and systemic therapy selection. Correlation with treatment response and recurrence risk, coupled with high specificity in nodal assessment [11], positions FAPI as a potential cornerstone in bladder cancer imaging. However, its limited performance in primary tumor assessment, due to urinary excretion, remains a barrier and highlights the need for optimized acquisition protocols [7].

Alongside new tracers, the integration of AI into PET/CT interpretation shows potential to standardize image assessment and reduce interobserver variability, although further validation in prospective, multicenter studies is still required [13]. In parallel, tumor-specific targets such as Nectin-4 and VEGF are emerging as promising avenues for molecular imaging and therapeutic selection. The strong correlation between ^68^Ga-N188 PET and IHC-determined Nectin-4 expression, with near-perfect specificity, supports its role as a companion diagnostic [15], while VEGF has shown moderate discriminatory ability. Conversely, EGFR and PSMA appear less suitable for bladder cancer imaging [14].

Although results with novel PET tracers are encouraging, most studies are still early-phase or pilot projects with small, heterogeneous cohorts, which limits generalizability and firm conclusions about clinical value. Standardized acquisition protocols, unified interpretation criteria, and head-to-head comparisons across tracers are largely absent. Robust cost-effectiveness data are also lacking. Large, prospective multicenter trials will be essential to validate these tracers, harmonize methodology, and support their integration into routine care.

At the cystoscopic level, WLC remains the standard but is hampered by a miss rate of up to 20% for flat and small lesions and incomplete TURBT rates approaching 50%. Both factors contribute to recurrence and progression [16,17,18,19]. Enhanced optical modalities such as fluorescence cystoscopy and NBI improve detection but face adoption barriers related to logistics, costs, and training [3,18,20]. Recent advances in AI are shifting this paradigm: deep learning models trained on cystoscopic images, particularly under blue-light conditions, now achieve sensitivities above 91% and specificities over 77% for NMIBC detection [22]. Beyond detection, AI tools assist in tumor margin delineation and segmentation [23,24,25,26,27,28,29,30,31,32], provide accurate grading comparable to experts [34,35], and even enhance real-time visualization during TURBT. These platforms reduce false positives and interobserver variability [36,37].

Despite its promise, AI-assisted cystoscopy faces major challenges: limited transparency of algorithms, variable image quality, and small, homogeneous training datasets that restrict generalizability and clinician trust. Data privacy and regulatory barriers hamper creation of large annotated repositories for external validation. Most evidence comes from retrospective, single-center cohorts, and algorithms often lose accuracy when applied to different populations or devices. Successful adoption will require standardized imaging protocols, hardware compatibility, urologist training, cost-effectiveness data, and prospective multicenter trials to demonstrate real-world benefit [33,38].

The field of theragnostics represents a further frontier, particularly relevant in urothelial carcinoma with its variable immune landscape. Immuno-PET enables the in vivo visualization of PD-L1, offering spatially and temporally resolved information beyond the static snapshot of IHC. Although promising for patient selection and monitoring immunotherapy response, no PD-L1 PET tracers have yet been tested in bladder cancer-specific trials. This gap underscores an important research gap. Meanwhile, antibody-drug conjugates targeting Nectin-4 and TROP-2, such as enfortumab vedotin and sacituzumab govitecan, have shown objective response rates of 31–44% in advanced or pretreated populations [53,54]. Expression patterns further support precision approaches: Nectin-4 is enriched in squamous and plasmacytoid variants, while PD-L1 predominates in sarcomatoid tumors [40,56].

Theragnostic strategies and immuno-PET are promising for precision oncology but face significant barriers, including intratumoral heterogeneity, resistance mechanisms, toxicity, cost, and the lack of validated biomarkers to guide patient selection [50,83]. Evidence is largely preclinical or from early-phase, single-center studies, with little bladder cancer-specific validation [42]. Antibody–drug conjugates show activity, but data are mostly limited to phase I/II trials with scarce real-world or long-term outcomes [53,54,55]. Regulatory hurdles for novel radiopharmaceuticals further delay translation, underscoring the need for standardized frameworks and robust, prospective, multicenter studies before routine use.

Parallel advances in biomarkers and liquid biopsy are transforming bladder cancer management toward precision medicine. Multiple analytes—including ctDNA, CTCs, RNAs (miRNAs, lncRNAs, circRNAs), proteins, exosomes, and metabolites—can be detected in blood and urine, providing minimally invasive tools for dynamic monitoring [57,59]. Clinically, biomarkers show promise for diagnosis, particularly in high-grade disease, and for follow-up, serving as adjuncts in NMIBC recurrence detection. They also have potential for prognosis, with markers such as p53, FGFR3, CYFRA 21-1, and ERCC1 being linked to disease progression and treatment response, although routine adoption still requires validation [81,84].

Among circulating biomarkers, ctDNA has emerged as one of the most informative. Its detection predicts recurrence and survival, often anticipating metastatic relapse by up to three months compared to imaging [84,85]. Urinary tumor DNA (utDNA) further complements ctDNA, with high sensitivity for minimal residual disease and the capacity to inform on tumor grade and stage, thereby supporting bladder-sparing strategies [80].

Radiogenomic approaches may strengthen biomarker-imaging integration, while clinical trials such as NEO-BLAST (combining utDNA, plasma ctDNA, imaging, and repeat TURBT) exemplify how multimodal strategies could redefine bladder preservation protocols [77,78]. Recent clinical trials have confirmed that combining imaging-based classifications with biomarker panels can improve treatment decisions in bladder cancer. For example, VI-RADS, an MRI scoring system that classifies the stage and extent of bladder cancer together with molecular profiling, has shown strong predictive value. The PURE-01 study is the most advanced illustration: in patients with muscle-invasive bladder cancer treated with neoadjuvant pembrolizumab, integrating VI-RADS scores (before and after treatment) with genomic profiling allowed the highly accurate prediction of pathological response (pT ≤ 1 N0) and survival [86]. Yet, tumor heterogeneity, limited sensitivity in early stages, and the absence of international consensus on optimal biomarker panels persist as key obstacles [87,88].

Biomarkers and liquid biopsy approaches hold great promise but are still mainly supported by small, observational, single-center studies, limiting generalizability. Differences in study design, sample processing and assay platforms complicate cross-study comparison [86]. Prospective multicenter trials with standardized protocols and predefined endpoints are still needed. Broader implementation also faces regulatory, cost and infrastructure barriers, as well as unequal access to advanced sequencing platforms and specialized training necessary for reproducibility and widespread adoption.

## 5. Conclusions

Bladder cancer management is entering a transformative era driven by molecular imaging, AI, theragnostics, and biomarker integration. Together, these innovations go beyond conventional visualization, enabling real-time, personalized guidance across diagnosis, treatment, and surveillance.

Next-generation PET tracers, such as FAPI, improve sensitivity for nodal and peritoneal disease, while immune-targeted probes like Nectin-4 and PD-L1 lay the foundation for theragnostic strategies. However, challenges remain in optimizing protocols for primary tumor visualization and expanding access to disease-specific tracers, requiring multicenter validation and cost-effectiveness analyses.

AI-based cystoscopic platforms are rapidly advancing, with deep learning systems achieving expert-level accuracy in detection, segmentation, and grading. These tools have the potential to standardize TURBT, reduce recurrence, and enhance surgical decision-making. Yet, issues of transparency, dataset diversity, and clinical validation still limit translation to practice, highlighting the need for pragmatic implementation studies across diverse healthcare systems.

Liquid biopsy and molecular biomarkers, particularly ctDNA, utDNA, and RNA panels, offer minimally invasive ways to track tumor dynamics and anticipate therapeutic responses earlier than imaging. Their integration with radiomics and multi-omic approaches could redefine risk stratification and patient selection. Still, heterogeneity and a lack of assay standardization remain major obstacles to routine adoption, emphasizing the importance of prospective, biomarker-driven clinical trials.

The convergence of imaging, AI, and molecular biomarkers represents a paradigm shift in uro-oncology. Clinical impact will depend on multicenter validation, international harmonization, and well-designed trials conducted in real-world settings.

Importantly, their combined use offers a holistic framework where complementary strengths of PET tracers, AI-assisted cystoscopy, liquid biopsy, and theragnostic approaches can advance precision oncology in bladder cancer. By bridging these domains, bladder cancer care can move toward true precision medicine—where therapeutic choices are guided by continuous, integrated molecular and imaging insights.

## Figures and Tables

**Table 1:** Global methods summary (own elaboration).

Clinical Question	Do Novel Molecular Imaging, AI-Assisted Cystoscopy, Theragnostics, and Molecular Biomarkers Improve Detection/Staging/Management vs. SoC in Patients with Bladder Cancer?
Population	(1) Humans; (2) Adults; (3) Patients with localized and/or advanced bladder cancer
Intervention	New PET tracers, PET/CT–PET/MRI, radiomics; AI-cystoscopy, NBI; theragnostics; ctDNA/utDNA, CTCs, multi-omics.
Comparison	Conventional imaging (CT, MRI, or [^18^F]FDG PET/CT); expert interpretation alone (for AI studies); standard clinical staging protocols. SoC: WLC ± BLC/NBI (no AI); urine cytology; EORTC/CUETO scores.
Outcomes	Primary: Sensitivity/specificity/AUC, T/N/M accuracy, change-in-management, minimal residual disease (ctDNA/utDNA), RFS/PFS/OS, therapy selection. Secondary: workflow, inter-reader, safety, cost-effectiveness, feasibility.
Study types	Prospective or retrospective clinical studies, systematic reviews, pilot feasibility studies
Databases searched	PubMed, Embase, Cochrane
Search keywords	Bladder cancer AND (PET/PET-CT/PET-MRI/FAPI/PSMA/radiomics) OR (cystoscopy AND AI/CAD/BLC/PDD/NBI) OR (theragnostic/radioligand) OR (biomarker/ctDNA/utDNA/CTC).
Manual search	Screening reference lists and journal issues by hand
Inclusion criteria	Adult BCa (NMIBC/MIBC) Diagnostic/staging & treatment response studies (2015–2025) Novel PET tracers vs. FDG/conventional imaging AI applied to PET/CT, MRI, cystoscopy/optical imaging Quantitative metrics (sens., spec., AUC, SUVmax) Biomarkers (ctDNA, CTCs, RNA, proteins) for Dx/prognosis/follow-up Imaging–biomarker integration (radiomics, mpMRI, multimodal) English/Spanish publications
Exclusion criteria	Reviews without patient-level data; case reports, editorials, abstracts without full text, opinion pieces

Abbreviations: AI = Artificial Intelligence; AUC = Area Under the Curve; BCa = Bladder Cancer; BLC = Blue Light Cystoscopy; CAD = Computer-Aided Detection; CT = Computed Tomography; CTCs = Circulating Tumor Cells; CUETO = Club Urológico Español de Tratamiento Oncológico; Dx = Diagnosis; EORTC = European Organisation for Research and Treatment of Cancer; FDG = Fluorodeoxyglucose; FAPI = Fibroblast Activation Protein Inhibitor; MRI = Magnetic Resonance Imaging; mpMRI = Multiparametric MRI; MIBC = Muscle-Invasive Bladder Cancer; NAC = Neoadjuvant Chemotherapy; NBI = Narrow Band Imaging; NMIBC = Non-Muscle-Invasive Bladder Cancer; OS = Overall Survival; PET = Positron Emission Tomography; PDD = Photodynamic Diagnosis; PFS = Progression-Free Survival; PSMA = Prostate-Specific Membrane Antigen; RFS = Recurrence-Free Survival; RNA = Ribonucleic Acid; SoC = Standard of Care; SUVmax = Maximum Standardized Uptake Value; T/N/M = Tumor/Node/Metastasis; TURBT = Transurethral Resection of Bladder Tumor; utDNA = Urinary Tumor DNA; WLC = White Light Cystoscopy.

**Table 2:** Summary of Key Clinical Studies on Molecular Imaging and PET Tracers in Bladder Cancer.

Authors	Year	Study Type	Main Objective	*n*	Additional Notes
Unterrainer et al. [6]	2022	Prospective	Detect nodal/hematogenous spread with ^68^Ga-FAPI-46 PET/CT	15	Improved detection over CT; useful for early metastatic staging
Novruzov et al. [8]	2022	Intra-individual comparison	Compare ^68^Ga-FAPI vs. ^18^F-FDG PET/CT in bladder cancer	8	Superior sensitivity in small nodes/peritoneum
Koshkin et al. [10]	2024	Prospective (NCT04621435)	Evaluate ^68^Ga-FAP-2286 PET/CT in urothelial carcinoma	21	Altered clinical decisions in 3 patients
Ortolan et al. [9]	2024	Narrative review	Discuss role of FAPI PET in urological cancers	–	Emphasizes diagnostic and theragnostic potential
Unterrainer et al. [11]	2024	Prospective	Assess locoregional LN staging with ^68^Ga-FAPI-46 PET/CT	18	Useful for surgical planning
Hagens et al. [7]	2024	Systematic review	Review diagnostic value of FAPI PET/CT in GU cancers	10	Encourages protocol optimization
Salminen et al. [12]	2018	Multicenter prospective (NCT01918592)	Evaluate ^11^C-acetate PET/MRI for staging and NAC response	22	Useful for monitoring response, not nodal staging
Girard et al. [13]	2023	Retrospective + ML	Machine learning model for LN metastasis detection on FDG PET/CT	87	Objective, reproducible tool
van der Fels et al. [14]	2022	Immunohistochemical study	Assess VEGF, EGFR, PSMA in nodal mets of bladder cancer	48 (LNs)	Only VEGF viable for imaging
Duan et al. [15]	2023	First-in-human (NCT05321316)	Evaluate ^68^Ga-N188 PET for Nectin-4 imaging	32	Promising diagnostic companion for ADCs

Abbreviations: ^18^F-FDG: FDG labeled with fluorine-18; ^68^Ga-FAPI: Gallium-68-labeled fibroblast activation protein inhibitor; ^68^Ga-FAP-2286: A specific gallium-68-labeled FAPI radiotracer; ^68^Ga-N188: Gallium-68-labeled radiotracer targeting Nectin-4; ^11^C-acetate: Carbon-11-labeled acetate; PET/CT-MRI: positron emission tomography/magnetic resonance imaging; NAC: neoadjuvant chemotherapy; LN: lymph node (plural: LNs = lymph nodes); SUVmax: maximum standardized uptake value; AUC: area under the curve; NPV: negative predictive value; IHC: immunohistochemistry; ADC: antibody–drug conjugate; ML: machine learning; TIS: Total immunostaining score; Se: Sensitivity; Sp: Specificity; GU: genitourinary; VEGF: Vascular Endothelial Growth Factor; EGFR: Epidermal growth factor receptor.

**Table 3:** Key Studies on AI-Assisted Cystoscopic Detection and Characterization in Urothelial Bladder Cancer.

Authors	Year	Study Type	*n*	Main Objective	AI/System	Arquitecture/Model	Image Technique
Ye et al. [26]	2025	Prospective diagnostic accuracy study	94	Evaluate AI (HRNetV2) for real-time bladder lesion detection during cystoscopy.	HRNetV2	CNN	WLC
Shkolyar et al. [4]	2025	Review study	-	Review enhanced imaging and AI use in cystoscopy and TURBT.	CystoNet-T	CNN	WLC
Li et al. [29]	2025	Retrospective diagnostic study	273	Develop an AI model (BTS-Net) for bladder tumor segmentation in imaging.	BTS-Net	Segmentation net	WLC
Kim et al. [28]	2024	Randomized retrospective clinical trial	5670	Validate AI software INF-M01 for detecting bladder tumors in cystoscopy images	INF-M01	CNN	WLC
Zhu et al. [21]	2024	Narrative review	-	Review machine learning/deep learning applications in urological cancer diagnosis and treatment.	Machine learning and deep learning	CNN	CT, MRI, US and cystoscopy images
Zlobina et al. [23]	2024	Observational comparative study	21	Assess spectroscopy for in vivo bladder cancer detection.	Machine learning	Classification algorithms	Fluorescence and diffuse reflectance spectroscopy
Hwang et al. [32]	2024	Single-center diagnostic classification and segmentation study.	772	Use VGG19 to classify bladder lesions and Deeplab v3+ to segment morphological types in cystoscopy images.	VGG19 + BeepLab v3+	CNN + segmentation net	WLC
Lee et al. [35]	2024	Prospective comparative diagnostic study	543	Identify the best CNN model (EfficientNetB0) for classifying bladder tumors in cystoscopy images.	EfficientNetB0	CNN	WLC
Zhao el al. [31]	2024	Diagnostic image-based segmentation study.	-	Propose NAFF-Net efficient segmentation of bladder tumors in endoscopic images	NAFF-Net	Segmentation net	WLC
Jia X et al. [36]	2023	Prospective pilot diagnostic study	67	Develop and test CystoNet-T for bladder tumor detection in WLC.	CystoNet-T	FPN	WLC
Chang et al. [37]	2023	Prospective pilot diagnostic study	50	Evaluate real-time CystoNet AI system integrated into live cystoscopy/TURBT video to detect bladder tumors during procedures.	CystoNet	CNN	WLC
Zhang et al. [30]	2023	Retrospective comparative study	-	Compare various attention mechanisms to improve bladder tumor segmentation performance in cystoscopic images.	Deep learning	ACS	WLC
Mutaguchi et al. [24]	2022	Retrospective diagnostic segmentation study.	120	Compare Dilated U-Net vs. standard U-Net for accurate bladder tumor segmentation in cystoscopy images.	U-net	Dilated convolution	WLC
Wu et al. [27]	2022	Multicenter prospective diagnostic study.	10,729	Evaluate CAIDS for detecting bladder cancer via cystoscopy, comparing its diagnostic accuracy against standard clinical assessment	CAIDS algorithm	Pyramid Scene Parsing Network (PSPNet)	WLC
Yoo et al. [34]	2022	Retrospective diagnostic study	10,991	Evaluate Mask R-CNN for tumor detection and grade prediction from WLC and narrow-band images.	Deep learning	Mask R-CNN	WLC + NBI
Ali et al. [22]	2021	Multicenter retrospective diagnostic classification study	216	Train and evaluate CNNs to classify malignancy, invasiveness, and grade from BLC.	Deep learning	CNN	BLC
Ikeda et al. [25]	2020	Retrospective diagnostic study.	109	Train a GoogLeNet-based CNN to distinguish tumor vs. normal bladder images	Deep learning	CNN	WLC
Shkolyar et al. [16]	2019	Multicenter prospective diagnostic study	95	Develop and validate CystoNet for automated bladder tumor detection in WLC.	CystoNet	CNN	WLC

Abbreviation: WLC: White-light cystoscopy; CNN: Convolutional neural network; BLC: Blue Light Cystoscopy; ACS: Attention mechanism based Cystoscopic images Segmentation model.; NBI: Narrow Band Images; FPN: Feature pyramid network.

**Table 4:** Clinical Studies on Biomarkers, Liquid Biopsy, and Imaging Integration in Bladder Cancer.

Authors	Year	Study Type	Main Objective	*n*	Key Statistical Findings	Additional Notes
Lodewijk et al. [59]	2018	Narrative review	Types, potential and applications of biomarkers in BCa.	-	Detect a much lower tumor burden	Generate significant anxiety and lead to patient overtreatment.
Zhang et al. [60]	2017	Meta-Analysis	The prognostic and diagnostic value of CTCs in BCa.	30	CTCs are an independent predictive indicator of poor outcomes.	-
Wan et al. [57]	2025	Review	Relevance of urine biomarkers	-	Advancements and limitations in BCa biomarkers	-
Lindskrog et al. [77]	2025	Review	Potential of ctDNA and CTC as biomarkers	-	Several studies have shown clinical potential	Combining biomarkers enhances diagnostic results.
Zulfiqqar et al. [61]	2025	Systematic Review and Meta Analysis	CTC positivity and recurrence and progression to NMIBC	5	CTCs enhance prognostic accuracy and therapeutic strategies in NMIBC,	CTC positivity after TURBT predicted recurrence
Liu et al. [62]	2022	Prospective	The value of CTCs and Ki-67 in prognosis	84	Ki-67 high expression associates with high postoperative CTC counts	-
Beije et al. [63]	2022	Multicenter Prospective Study	CTCs to drive the use of NAC in MIBC	273	CTC+ who received NAC survived longer	Negative CTCs alone does not justify withholding NAC.
Grosso et al. [65]	2025	Systematic Review	Prognostic role of ctDNA in the perioperative of MIBC	8	ctDNA+ pre and postoperative associates with poor clinical outcomes.	ctDNA could guide NAC management.
Liu et al. [66]	2024	Meta Analysis	Prognostic significance of ctDNA in BCa	1275	Strong association of ctDNA dynamic change with survival outcomes.	Clinical utility of ctDNA in prognosis
Li et al. [69]	2024	Study	Value of miRNA-143 in the early detection of BCa	4054	Sensitivity and specificity were 0.80 and 0.85	Coupled with miR-100, it showed better diagnostic power
Vandekerkhove et al. [72]	2017	Study	ctDNA to profile the tumor genome in real-time in BCa	51	ctDNA provides a practical and cost-effective snapshot of driver gene status in metastatic BCa	95% of metastatic patients harboring alterations to TP53, RB1, or MDM2, an 70% in ARID1
Moisoiu et al. [74]	2022	Retrospective cohort	Combined miRNA and SERS for diagnosis and molecular stratification	31	miRNA profiling synergizes with SERS	Discriminate between high-grade and low-grade tumors and between luminal and basal types.
O’Sullivan et al. [78]	2025	Review	Radiomics-based nomogram to predict oncological outcomes in BCa		Several studies demonstrate the predictive potential of radiogenomic	Further studies are required to validate the results
Shohdy et al. [79]	2022	Prospective study	ctDNA as a dynamic tool for changes in the VAF of genomic alterations	53	Serial ctDNA analysis predicts disease radiologic progression	-
Lee et al. [80]	2025	Review	The current state of utDNA as a marker of BCa	-	utDNAl improves on most of stages of detection, treatment, and monitoring	-
Matuszcz et al. [81]	2020	Review	Find the one urine biomarker with the best specificity and sensitivity		p53, FGFR3, CYFRA 21-1, and ERCC1 clinical applications	-
Yu Lu et al. [82]	2025	Meta-Analysis	Predictive value of ctDNA detection for disease progression and metastasis risk	9	ctDNA demonstrates some application value in the prognostic, recurrence and survival.	Not enough to replace traditional assessment methods

Abbreviation: BCa = Bladder Cancer; ctDNA = Circulating Tumor DNA; CTCs = Circulating Tumor Cells; NAC = Neoadjuvant chemotherapy; DFS = Disease-Free Survival; HR = Hazard Ratio; IHC = Immunohistochemistry; lncRNA = Long Non-Coding RNA; miRNA = MicroRNA; mRNA = Messenger RNA; MRD = Minimal Residual Disease; NAC = Neoadjuvant Chemotherapy; NMIBC = Non-Muscle-Invasive Bladder Cancer; MIBC = Muscle-Invasive Bladder Cancer; OS = Overall Survival; RFS = Recurrence-Free Survival; SERS = Surface-Enhanced Raman Spectroscopy; utDNA = Urinary Tumor DNA.

## Data Availability

The original contributions presented in this study are included in the article; further inquiries can be directed to the corresponding author.

## Abbreviations

The following abbreviations are used in this manuscript:

AI	Artificial Intelligence
CT	Computed tomography
PET	Positron emission tomography
MRI	Magnetic resonance imaging
AUC	Area under curve
BCa	Bladder Cancer
BLC	Blue Light Cystoscopy
CAD	Computer-Aided Detection
CTCs	Circulating Tumor Cells
ctDNA	Circulating Tumor DNA
CUETO	Club Urológico Español de Tratamiento Oncológico
Dx	Diagnosis
DFS	Disease-free Survival
EORTC	European Organisation for Research and Treatment of Cancer
FDG	Fluorodeoxyglucose
FAPI	Fibroblast Activation Protein Inhibitor
IoU	Intersection over Union
mpMRI	Multiparametric magnetic resonance imaging
MIBC	Muscle-Invasive Bladder Cancer
NAC	Neoadjuvant Chemotherapy
NBI	Narrow Band Imaging
NMIBC	Non-Muscle-Invasive Bladder Cancer
OS	Overall Survival
PDD	Photodynamic Diagnosis
PFS	Progression-Free Survival
PSMA	Prostate-Specific Membrane Antigen
RFS	Recurrence-Free Survival
RNA	Ribonucleic Acid
SoC	Standard of Care
SUVmax	Maximum Standardized Uptake Value
T/N/M	Tumor/Node/Metastasis
TURBT	Transurethral Resection of Bladder Tumor
utDNA	Urinary Tumor DNA
WLC	White Light Cystoscopy
^18^F-FDG:	FDG labeled with fluorine-18
^68^Ga-FAPI	Gallium-68-labeled fibroblast activation protein inhibitor
^68^Ga-FAP-2286	A specific gallium-68-labeled FAPI radiotracer
^68^Ga-N188	Gallium-68-labeled radiotracer targeting Nectin-4
^11^C-acetate	Carbon-11-labeled acetate
NPV	negative predictive value
IHC	Immunohistochemistry
ADC	antibody–drug conjugate
ML	Machine learning
TIS	Total immunostaining score
Se	Sensitivity
Sp	Specificity
GU	genitourinary
CNN	Convolutional neural network
ACS	Attention mechanism based Cystoscopic images Segmentation model
FPN	Feature pyramid network
HR	Hazard Ratio
lncRNA	Long Non-Coding RNA
miRNA	MicroRNA
mRNA	Messenger RNA
MRD	Minimal Residual Disease
SERS	Surface-Enhanced Raman Spectroscopy
EGFR	Epidermal growth factor receptor
VEGF	Vascular Endothelial Growth Factor
BCG	Bacillus Calmette–Guérin
